# Circulating Th1, Th2, Th17, Treg, and PD-1 Levels in Patients with Brucellosis

**DOI:** 10.1155/2019/3783209

**Published:** 2019-08-06

**Authors:** Rongjiong Zheng, Songsong Xie, Qiong Zhang, Ling Cao, Shaniya Niyazi, Xiaobo Lu, Lihua Sun, Yan Zhou, Yuexin Zhang, Kai Wang

**Affiliations:** ^1^Department of Infectious Diseases, The First Affiliated Hospital of Xinjiang Medical University, Urumqi, Xinjiang 830054, China; ^2^Department of Clinical Laboratory, The First Affiliated Hospital of Xinjiang Medical University, Urumqi, Xinjiang 830054, China; ^3^Department of Medical Engineering and Technology, Xinjiang Medical University, Urumqi, Xinjiang 830011, China

## Abstract

*Brucella* is an intracellular infection bacterium; the pathogenesis of *Brucella* and chronicity of infection may be related to the immune response of T cells. T lymphocytes mainly participate in cellular immune response. The extent of different T cell subsets imbalanced and their function dysregulated in patients with brucellosis remain not explicit. We grouped patients at different stages (acute, chronic, and convalescent). The frequencies of Th1, Th2, Th17, Treg, and PD-1 (programmed cell death protein 1) in peripheral blood were examined by flow cytometry, and the expressions of T lymphocyte cytokines in serum were detected by cytometric bead array. Th1, Th17, and Treg cell immunity was predominant in the acute stage, while Th2, Th17, and Treg cell immunity was predominant in the chronic stage. The expressions of PD-1 on CD4+ and CD8+ T lymphocytes were significantly different in acute and chronic patients. The percentages of Th1 cells in convalescent patients were still higher than those in healthy controls within one year after withdrawal. The expression of T lymphocyte cytokines in serum was different in patients at different stages. These results indicate that peripheral T lymphocyte immunity was involved in patients with brucellosis and represents a target for the preclinical and clinical assessment of novel immunomodulating therapeutics. The patients' immune function had not completely recovered in a short period of time during convalescence, so long-term follow-up of convalescent patients is needed.

## 1. Introduction

Brucellosis is considered to be a zoonotic disease with a high disability rate and great harm, which seriously threatens public health. *Brucella* is currently considered to be a model for immunological research against intracellular bacterial infection. In 1958, Holland and Pickett first demonstrated that *Brucella* was widely replicated in mouse macrophages [1].

Host protection against *Brucella* depends on cell-mediated immune response, mainly including activated APC, Th1 cells, and CD8+ CTL. On the other hand, *Brucella* has developed strategies to avoid congenital and adaptive immune responses, aiming to establish intracellular niches for long-term survival and replication [2–5]. Th1 and Th2 cells are the earliest known subsets of CD4+ T lymphocytes [6, 7], and Th1 plays an important role in bacterial infection by secreting cytokines such as IFN-gamma and IL-2. It can eliminate bacterial infection, especially for intracellular bacteria. Th1 generally plays a role in the early stage of infection and plays a key role in the host's resistance to pathogens, especially for intracellular bacterial infections such as *Mycobacterium tuberculosis*. The immune function of Th2 cells in pathogenic infection is mainly to induce humoral immunity. There is a regulatory relationship between Th17 cells and the induction and differentiation of Th1 cells, Th2 cells, and Treg cells. The growth and development of Th17 cells are negatively regulated by IFN-gamma and IL-4. Once mature, Th17 cells can resist the inhibition of IFN-gamma and IL-4. Th17 cells are different from Th1 and Th2 inflammatory cell lines, which make up for the deficiency of the Th1-/Th2-mediated effect mechanism. CD4+ regulatory T cells are considered as a kind of T lymphocyte subset with mature negative immunoregulatory function. Treg cells inhibit the differentiation and proliferation of T lymphocytes [8, 9].

In our previous studies [10], we systematically evaluated the changes of T lymphocyte subsets in the peripheral blood of patients with brucellosis by meta-analysis. The results showed that the frequency of CD4+ T lymphocytes and the ratio of CD4/CD8 cells in the peripheral blood of patients with brucellosis were significantly lower than those of healthy controls, and the frequency of CD8+ T lymphocytes was higher than that of healthy controls. Our study has some limitations: (1) There are few studies on the immune function of *Brucella* patients. We have screened only 8 out of more than 5,000 articles. There are few or no reports on the expression of Th1, Th2, Th17, and Treg-related T cell subsets and PD-1 in patients with brucellosis. (2) Most of the reported studies did not compare acute, chronic, and convalescent *Brucella* patients.

The specific mechanism of the immune function after *Brucella* infection is not clear. It is presumed that *Brucella* infection is prone to chronicity mainly because it can evade the immune clearance of the body, and the disorder of T lymphocyte subsets is the main reason why the human body cannot clear *Brucella* and it becomes chronic. Peripheral blood T cell subsets are important indicators to reflect the immune status of patients. Clarifying the immune status of patients at different stages of *Brucella* infection can provide research direction for the more timely and effective reduction of the chronicity of *Brucella* infection in patients.

T lymphocyte is composed of different cell subsets, in which the number and function of different cell subsets determine the immune status of the host. T lymphocyte subsets form a complex immune network in vivo by secreting a variety of cytokines. The normal immune function of the human body is maintained with a certain balance ratio between different cells. Once this balance is broken, it can lead to immune disorders in the body [11, 12].

Immune damage plays an important role in the occurrence and development of the disease, especially in the chronic process. T lymphocyte plays a key role in the elimination of pathogens after *Brucella* infection. Programmed death molecule 1, with a molecular weight of about 50-55 kDa, belongs to the CD28 superfamily, also known as CD279, which mediates negative costimulatory signals of T lymphocytes and inhibits proliferation, differentiation, and cytokine secretion of T lymphocytes. Many studies have shown that PD-1 weakens the host's ability to clear up pathogens in viral, bacterial, and tuberculosis infectious diseases [13–16].

The current literatures on the research of the expression of Th1, Th2, Th17, and Treg-related T cell subsets and PD-1 in brucellosis patients at different stages (acute, chronic, and convalescent stages) are rare or unreported. In order to further clarify the role of immune function in the pathogenesis of brucellosis, we explored the characteristics of the immune response of T cell subsets and related cytokines and PD-1 in patients with brucellosis and provided theoretical basis for the further study of the immunological pathogenesis of *Brucella* infection.

## 2. Methods

### 2.1. Subjects

125 cases of brucellosis in inpatient and outpatient departments of the First Affiliated Hospital of Xinjiang Medical University from March 2017 to December 2017 were collected. There were 45 patients of Han nationality, 65 patients of Kazakh nationality, 5 patients of Hui nationality, 7 patients of Uygur nationality, and 3 patients of Mongolian nationality. Among them, 47 cases were in the acute stage (age: 18-70 years; average age: 44.04 ± 11.26 years; 34 cases were male and 13 cases were female), 43 cases were in the chronic stage (age: 23-69 years; average age: 45.30 ± 12.24 years; 31 cases were male and 12 cases were female), 35 cases were in the convalescent stage (age: 18-72 years; average age: 43.66 ± 14.76 years; 25 cases were male and 10 cases were female), and 30 were healthy controls (age: 25-70 years; average age: 42.3 ± 13.9 years; 21 cases were male and 9 cases were female).

The criteria for diagnosis and clinical staging of brucellosis refer to the guidelines issued by China in 2012 for the diagnosis and treatment of brucellosis: (1) acute phase—the duration of disease was less than 6 months; (2) chronic phase—the disease had not recovered for more than 6 months; and (3) convalescence phase—at present, there is no definition of convalescent period in China and WHO guidelines. In this study, patients who completed the regular course of treatment, whose clinical symptoms disappeared, and whose laboratory inflammation markers returned to normal were regarded as convalescent patients.

### 2.2. Inclusion Criteria

Patients who met the diagnostic criteria for brucellosis guidelines issued in 2012 were included in the study.

### 2.3. Exclusion Criteria

The exclusion criteria are as follows: (1) patients with a history of serious diseases or dysfunction of other systems (cardiovascular, nervous, respiratory, liver, kidney, etc.); (2) patients with a history of tumor or immune system disorders; (3) patients who used immunosuppressive drugs, corticosteroids, and immunomodulators for a long time or nearly three months; and (4) patients with an immunity disorder.

This study was approved by the Ethics Committee of the First Affiliated Hospital of Xinjiang Medical University. All patients and healthy controls had signed informed consent forms.

### 2.4. Reagents

Human antibodies anti-human CD4, anti-human CD25, anti-human CD127 (IL-7R*α*), anti-human IL-17A, anti-human IL-4, and anti-human IFN-*γ* were all from BioLegend, USA [17]. CBA kits for Human Th Cytokine Panel (13-plex) were from BioLegend, USA [18]. Stimulants, fixed membrane breaking agents, and RBC lysates were all from Becton, Dickinson, and Company, USA.

### 2.5. Flow Cytometric Analysis of CD4+ Treg Subsets in Whole Blood

100 *μ*L anticoagulant peripheral blood was taken, and surface-labeled antibodies CD4, CD25, and CD127 were added. Simultaneously, the same type of control tube was set up and incubated for 30 minutes without light. Hemolysin was added to lyse red blood cells, the solution was centrifuged at 300 g for 5 minutes, and then the supernatant was discarded. After 2 mL cell lotion was added, the solution was centrifuged at 500 g for 5 minutes, and then the supernatant was discarded. 500 *μ*L of cell washing solution was added, mixed evenly, and then detected on a machine.

### 2.6. Flow Cytometric Analysis of Th1, Th2, and Th17 Subsets in Whole Blood

200 *μ*L anticoagulant peripheral blood was added into a 96-well culture plate, and a combined PMA+ionomysin+BFA stimulant was added, mixed evenly, and then put into a 37 degree 5% CO_2_ incubator for 4 hours. 200 *μ*L of whole blood was taken out from the flow tube, and the surface-labeled antibody CD4 was added. At the same time, the same type of control tube was set up. The whole blood was mixed and hatched for 20 minutes without light. Hemolysin was added to lyse red blood cells, and the supernatant was discarded after centrifugation at 500 g for 5 minutes. 2 mL cell lotion was added, the solution was centrifuged at 500 g for 5 minutes, and then the supernatant was discarded. 0.5 mL membrane solution was added, mixed evenly, and incubated at room temperature without light for 50 minutes. 2 mL cell lotion was added, the solution was centrifuged at 500 g for 5 minutes, and then the supernatant was discarded again. Intracellular antibodies (IFN-*γ*, IL-4, and IL-17A) were added, and the corresponding homologous control antibodies were added to the homologous control tube. The cells were incubated for 50 minutes without light exposure. 2 mL cell lotion was added, the solution was centrifuged at 500 g for 5 minutes, and then the supernatant was discarded again. 500 *μ*L cell washing solution was added, mixed evenly, and then detected on a machine.

### 2.7. Flow Cytometric Analysis of PD-1 on CD4+ and CD8+ T Lymphocytes in Whole Blood

5 *μ*L CD3, 20 *μ*L CD4, 5 *μ*L CD8, and 5 *μ*L PD-1 surface-labeled antibodies were added into 100 *μ*L whole blood. After mixing for 30 minutes, hemolysin was added to lyse red blood cells, the solution was centrifuged at 300 g for 5 minutes, and then the supernatant was discarded. After 2 mL cell lotion was added, the solution was centrifuged at 500 g for 5 minutes, and then the supernatant was discarded. 500 *μ*L cell washing solution was added, mixed evenly, and then detected on a machine.

### 2.8. Cytometric Bead Analysis of T Lymphocyte Cytokines

The expression levels of IL-5, IL-13, IFN-gamma, IL-2, IL-17A, IL-17F, and IL-4 in the peripheral serum of patients with brucellosis and healthy controls were detected using the Human Th Cytokine Panel (13-plex) Kit, according to the manufacturers' instructions.

### 2.9. Statistical Analysis

R version 3.4.2 was used to analyze the data. If the data obeyed normal distribution and homogeneity test of variance, a *t*-test of independent samples was used to compare the data between the two groups. If it failed to conform to normal distribution or homogeneity of variance, the Mann-Whitney *U* test was used to test the rank sum of two independent samples. The Kruskal-Wallis rank sum test was used to compare the data between groups, and the LSD method was used to compare the data between two groups. For the correlation analysis between the parameters of each index, the data conformed to the normal distribution using the Pearson method for correlation analysis, the Spearman method was used for nonnormal data, and the range of correlation coefficient *r* was [−1, +1]. A *P* value less than 0.05 had statistical significance.

## 3. Results

### 3.1. Subjects

#### 3.1.1. Contact History

Of the 125 brucellosis patients, 110 (88%) had a clear contact history, including 15 patients (12%) who had a history of veterinary occupational exposure, 8 patients (6.4%) who were animal skin and meat processors, and 87 patients (70%) who were cattle and sheep breeders with or without contact or eating uncooked beef, mutton, and dairy products; only 15 patients (12%) did not have a contact history.

#### 3.1.2. Clinical Manifestations

Among the 125 patients with brucellosis, 47 were in an acute stage (the duration ranged from 1 week to 6 months), 43 were in a chronic stage (the duration ranged from 7 months to 2 months), and 35 were in a convalescent stage (the duration of treatment ended from 2 months to 2 years). The clinical characteristics of patients at different clinical stages were analyzed. The results showed that there were some differences in clinical manifestations between acute and chronic patients. The main clinical manifestations of acute stage patients were fever, fatigue, muscle and joint pain, and hyperhidrosis. The main clinical manifestations of chronic stage patients were fatigue, fever, and joint pain. Few patients showed sequelae after clinical cure, such as fatigue syndrome and intermittent joint pain, and few patients had hyperhidrosis (Table 1).

#### 3.1.3. Laboratory Examinations

The laboratory indicators of all patients were analyzed. All patients were positive for erythrocyte plate test and tube agglutination test. The titers of antibodies in acute patients were 1 : 100-1 : 800, in chronic patients 1 : 50-1 : 800, and in convalescent patients 1 : 50-1 : 200. In acute stage, the positive rate of blood culture was relatively low, and there were no positive results in blood culture in patients at chronic stage. The abnormal rate of erythrocyte sedimentation and the C reactive protein levels in the chronic phase were lower than those in the acute phase. *Brucella* infection could cause abnormal results in laboratory examinations of multiple organ systems, such as the liver, spleen, and lymph nodes, which are the most common, mainly manifested as low trilineage, abnormal liver function, pulmonary inflammation, positive urinary protein, etc. (Table 2).

### 3.2. Results of Flow Cytometry Test

#### 3.2.1. Percent Proportion of PD-1 on CD4+ and CD8+ T Lymphocytes

**Figure 1:**
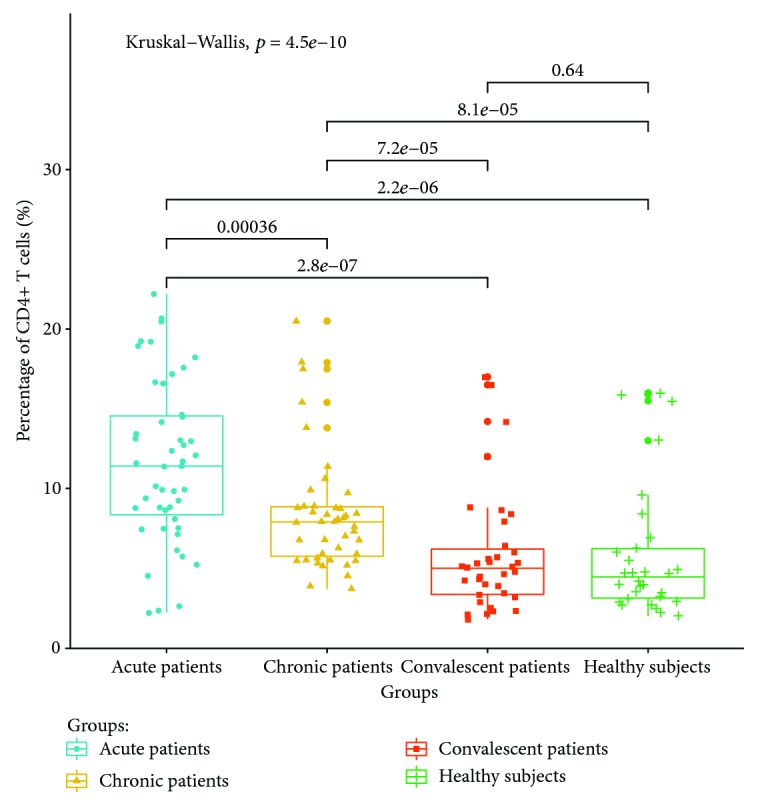
Expression ratio of PD-1 on the CD4+ T cell surface in the peripheral blood of different groups.

**Figure 2:**
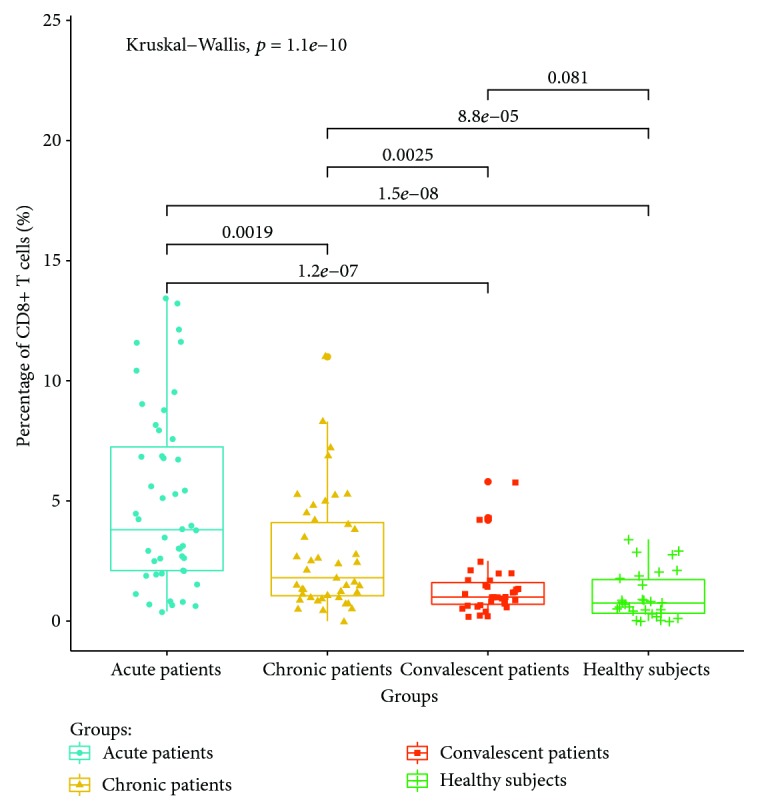
Expression ratio of PD-1 on the CD8+ T cell surface in the peripheral blood of different groups.

The expressions of PD-1 on CD4+ and CD8+ T cells in the acute brucellosis group were significantly higher than those in the chronic brucellosis group (*P* < 0.05). The expressions of PD-1 on CD4+ and CD8+ T cells in the chronic brucellosis group were higher than those in convalescent patients and healthy controls (*P* < 0.05). There were no significant differences in the expression of PD-1 on CD4+ and CD8+ T cells between convalescent patients and healthy controls (*P* > 0.05). The expression ratios of PD-1 on the CD4+ T and CD8+ T cell surface are shown in Figures 1 and 2, respectively.

#### 3.2.2. Percent Proportion of T Lymphocyte Subsets

**Figure 3:**
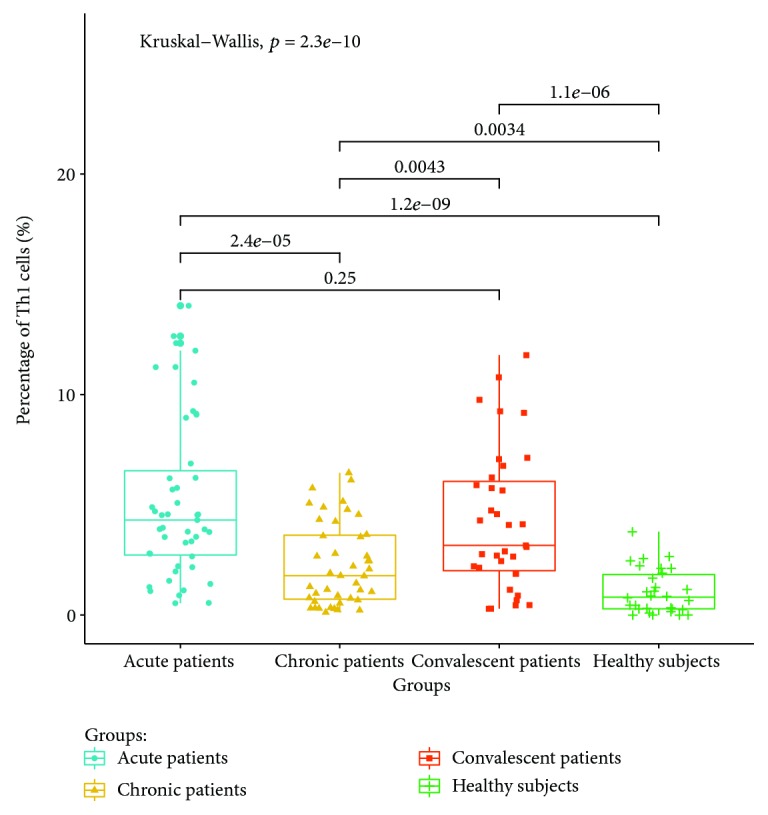
Expression ratio of Th1 in the peripheral blood of different groups.

**Figure 4:**
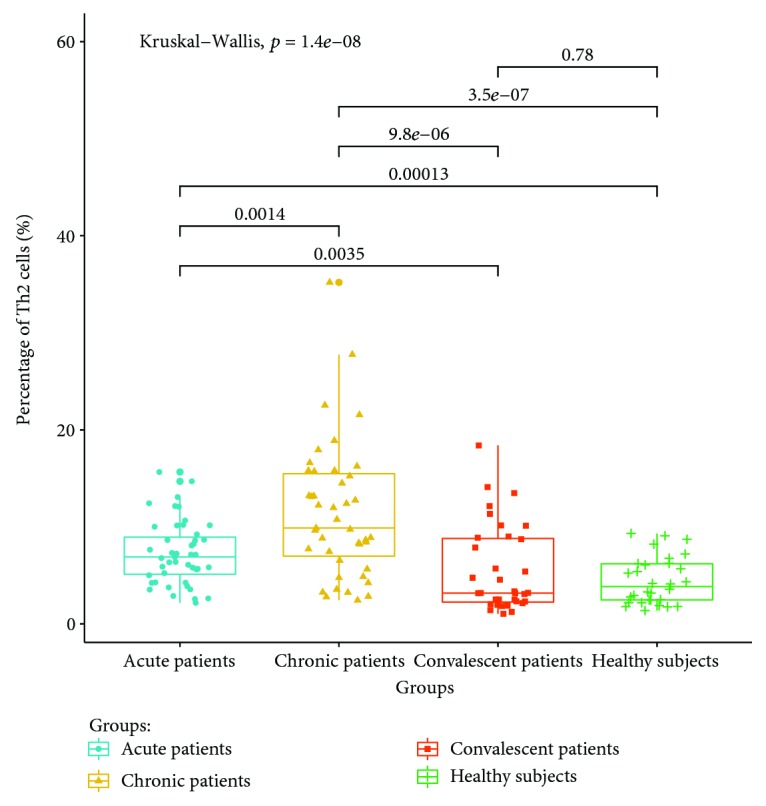
Expression ratio of Th2 in the peripheral blood of different groups.

**Figure 5:**
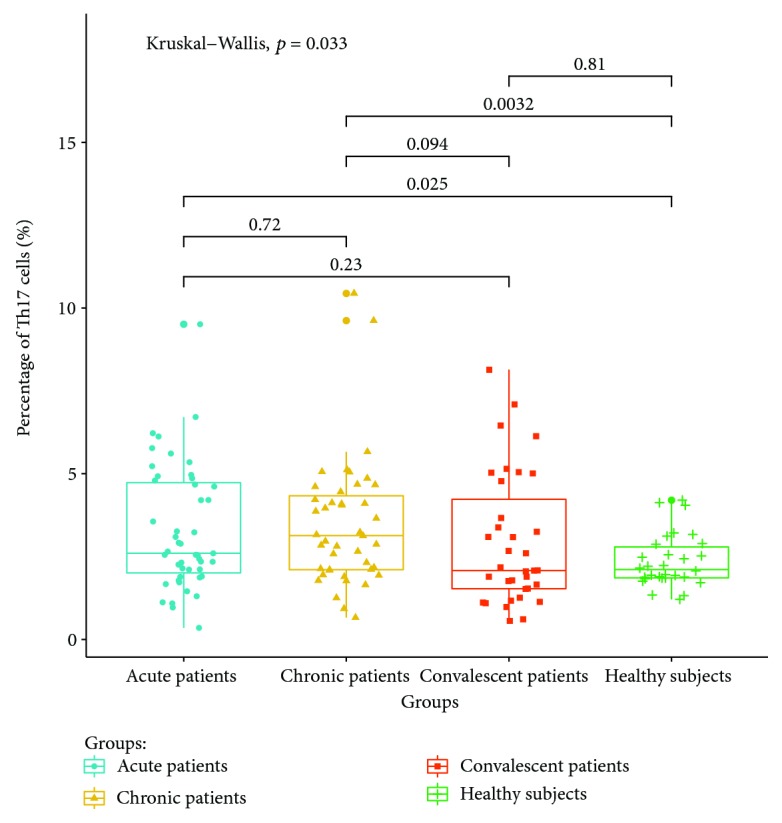
Expression ratio of Th17 in the peripheral blood of different groups.

**Figure 6:**
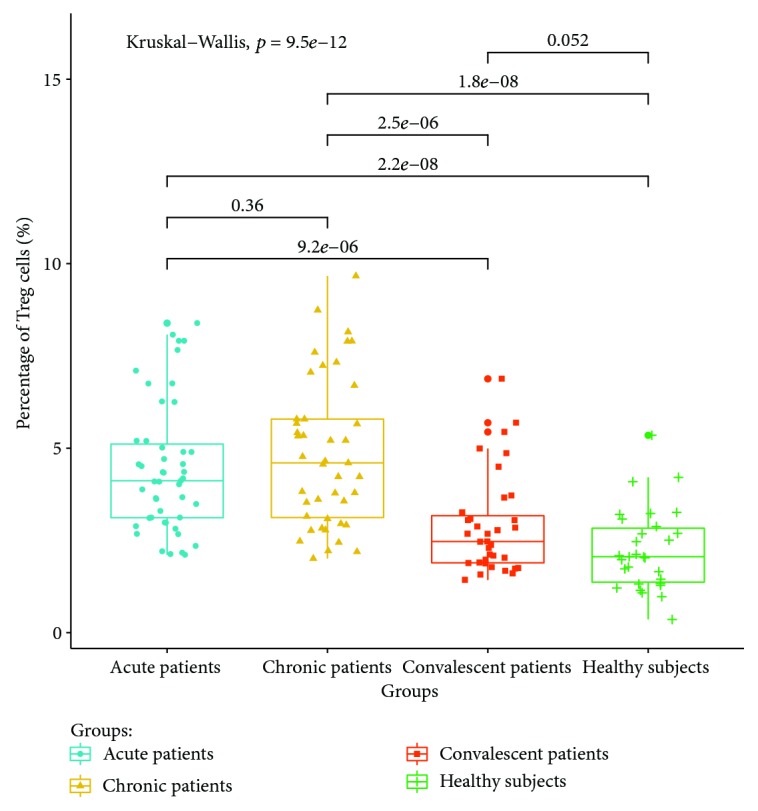
Expression ratio of Treg in the peripheral blood of different groups.

The expressions of T lymphocyte subsets in the peripheral blood of patients with acute, chronic, and convalescent brucellosis and healthy controls were detected by flow cytometry. The results showed that (1) CD4+ IFN-gamma (Th1) lymphocyte expressions in the peripheral blood of patients in the acute stage were significantly higher than those of patients in the chronic, convalescent, and healthy control groups (*P* < 0.05). The percentage of Th1 cells in the peripheral blood of chronic patients was higher than those of convalescent patients and healthy controls (*P* < 0.05). The percentage of Th1 cells in the peripheral blood of convalescent patients was higher than that of healthy controls (*P* < 0.05). The expression ratio of Th1 is shown in Figure 3. (2) The percentage of CD4+ IL-4 (Th2) lymphocytes in peripheral blood was the highest in chronic patients, which was significantly higher than those in the acute, convalescent, and healthy control groups. The percentage of CD4+ IL-4 (Th2) lymphocyte expression in acute patients was higher than those in the convalescent and healthy control groups (*P* < 0.05). There were no significant differences between convalescent patients and healthy control groups (*P* > 0.05). The expression ratios of Th1 are shown in Figure 4. (3) The percentage of CD4+ IL-17A (Th17) lymphocytes had no significant differences between acute and chronic patients (*P* > 0.05), but the expression of CD4+ IL-17A was higher than that of healthy controls (*P* < 0.05). There were no significant differences in the percentage of Th17 lymphocyte expression between convalescent patients and healthy controls (*P* > 0.05). The expression ratios of Th1 are shown in Figure 5. (4) The percentage of CD4+ CD25+ CD127LOW (Treg) cells in the peripheral blood of patients at the acute and chronic stages had no differences (*P* > 0.05), and were significantly higher than those of patients in the convalescent and healthy control groups (*P* < 0.05). There were no significant differences in the expression of CD4+ CD25+ CD127LOW (Treg) cells between patients from the convalescent group and patients from the healthy control group (*P* > 0.05). The expression ratio of Th1 is shown in Figure 6.

#### 3.2.3. Detection of Peripheral Blood T Lymphocyte Subsets in Convalescent Patients at Different Stages of Treatment

**Figure 7:**
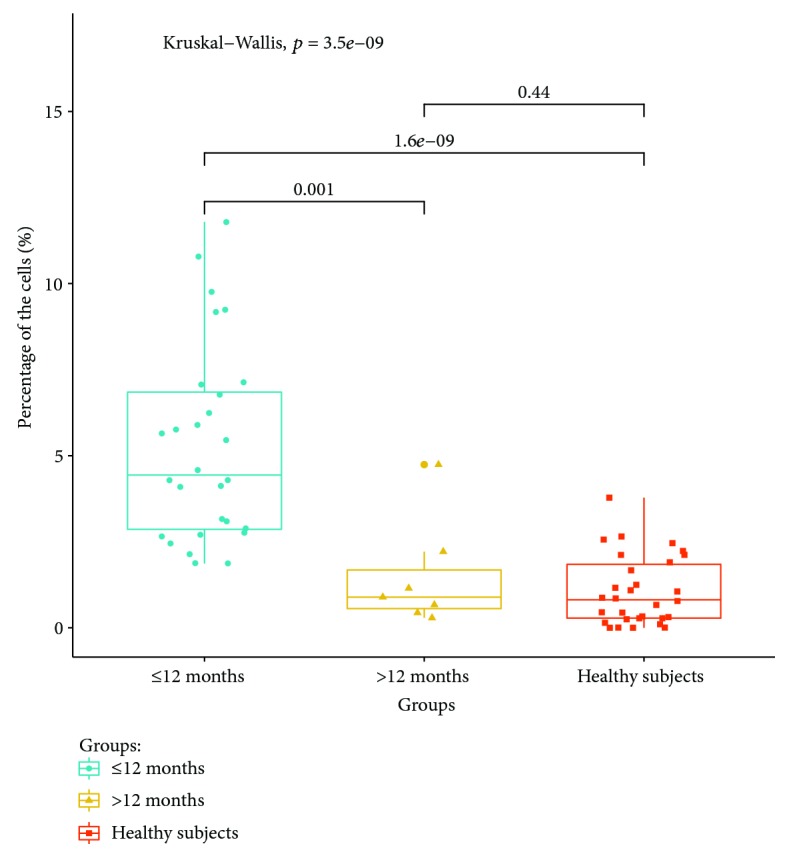
Expression ratio of Th1 in convalescent patients at different stages of treatment.

The expression ratios of T lymphocyte subsets in convalescent patients with drug withdrawal less than 12 months and in those with drug withdrawal more than 12 months were compared and analyzed. The results showed that the expression ratio of Th1 cells in peripheral blood was significantly higher in convalescent patients with drug withdrawal over 12 months (*P* < 0.05). The percentage of Th1 cells in peripheral blood in 12 months was still higher than that in the healthy control group (*P* < 0.05). There were no significant differences in the percentage of Th1 expression between convalescent patients and the healthy control group (*P* > 0.05). The expression ratio of Th1 in convalescent patients at different stages of treatment is shown in Figure 7. Th2, Th17, and Treg were expressed in the peripheral blood of convalescent patients in two periods (*P* > 0.05). There were no significant differences in proportions (*P* > 0.05).

#### 3.2.4. Correlation between T Lymphocyte Subsets and Withdrawal Time for Convalescent Patients

In this study, 35 patients who finished standard antimicrobial therapy had no clinical symptoms and normal laboratory tests. The withdrawal time ranged from 2 months to 24 months. Among them, 28 patients (80%) finished treatment within 12 months, and 7 patients finished treatment over 12 months. The correlation between the percentage of T lymphocyte in peripheral blood and the time of drug withdrawal showed that the percentage of Th1 cell expression in the peripheral blood of convalescent patients was negatively correlated with the time of drug withdrawal (*P* < 0.05). Correlation between T lymphocyte subsets and withdrawal time in convalescent patients is shown in Table 3.

#### 3.2.5. T Lymphocyte Cytokine Levels in Peripheral Serum

**Figure 8:**
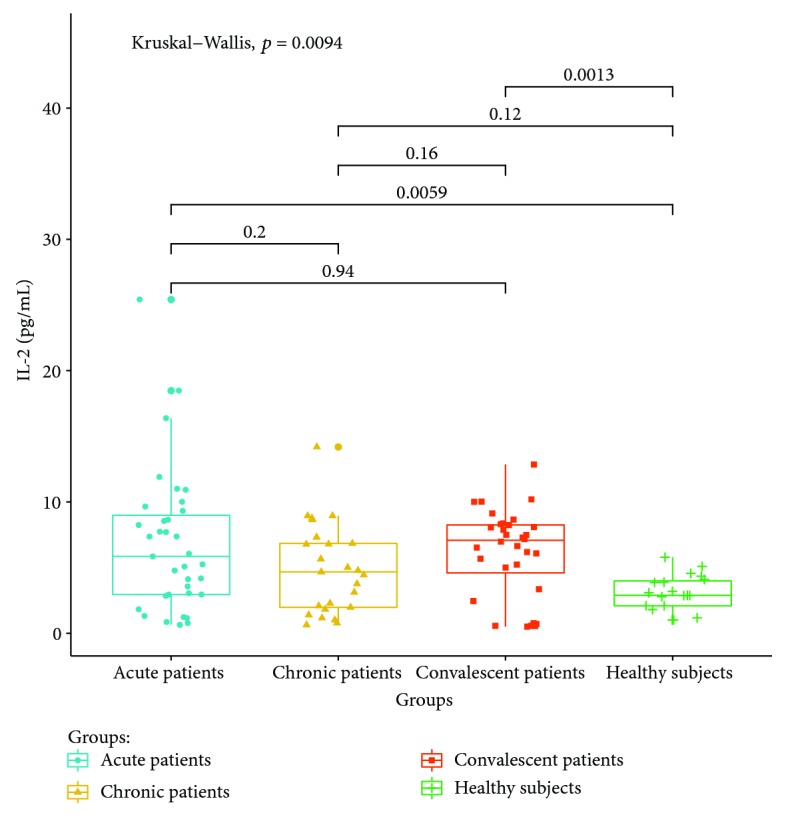
Expression of IL-2 in the peripheral blood of different groups.

**Figure 9:**
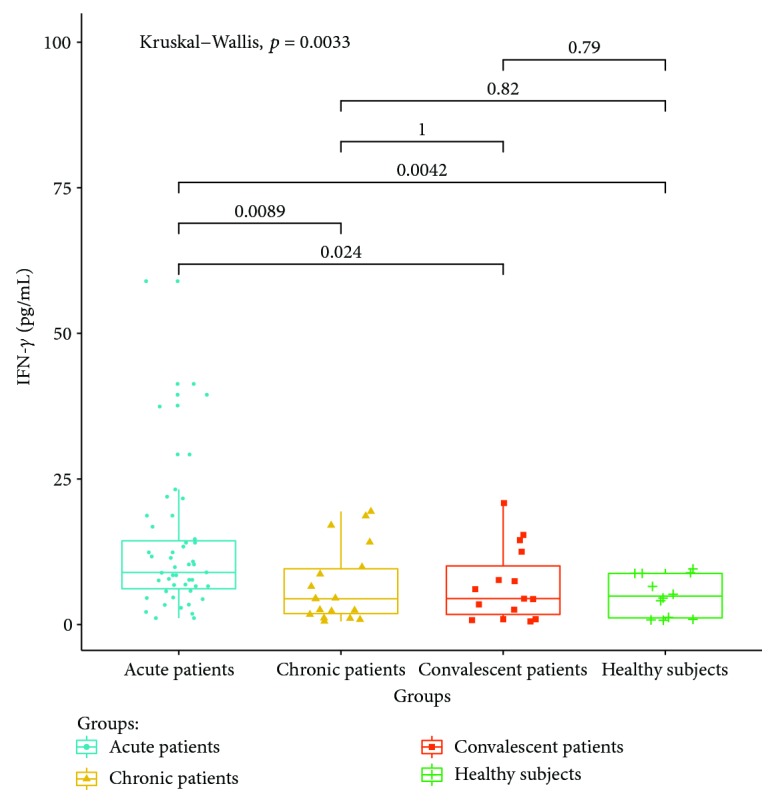
Expression of IFN-*γ* in the peripheral blood of different groups.

**Figure 10:**
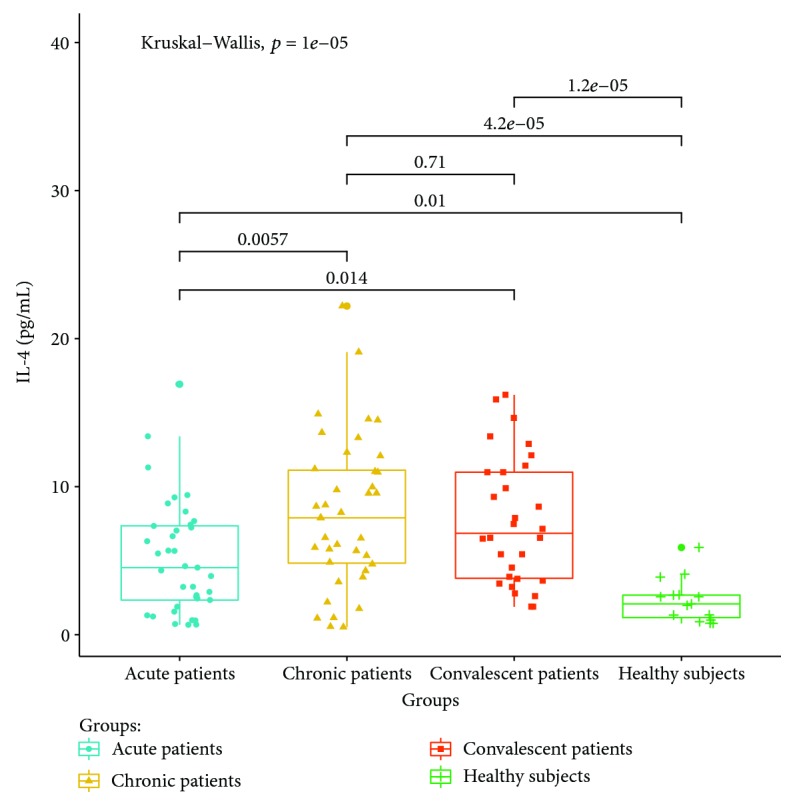
Expression of IL-4 in the peripheral blood of different groups.

**Figure 11:**
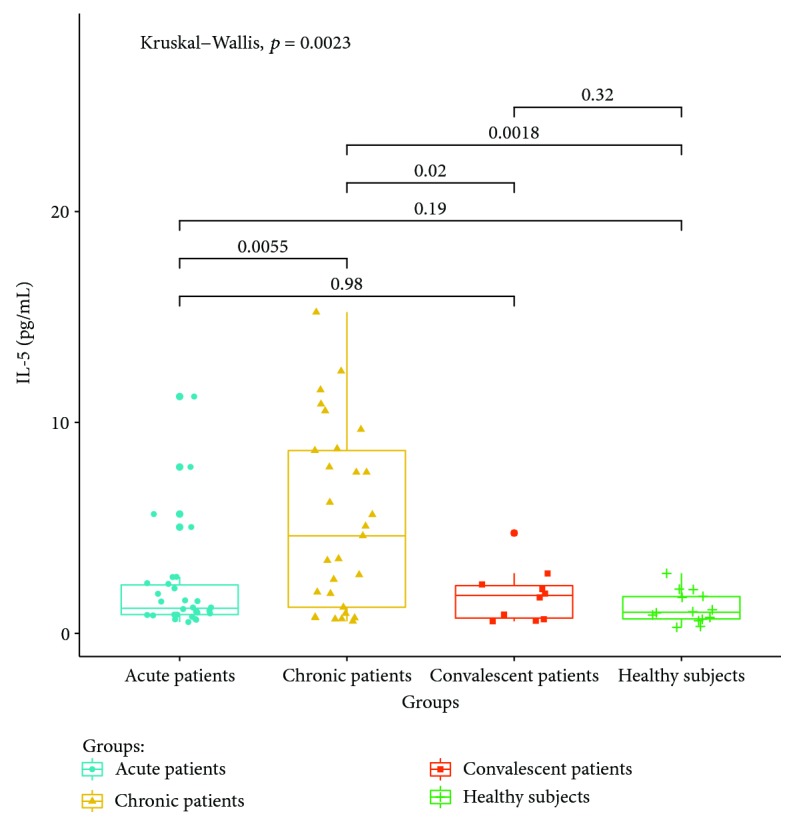
Expression of IL-5 in the peripheral blood of different groups.

**Figure 12:**
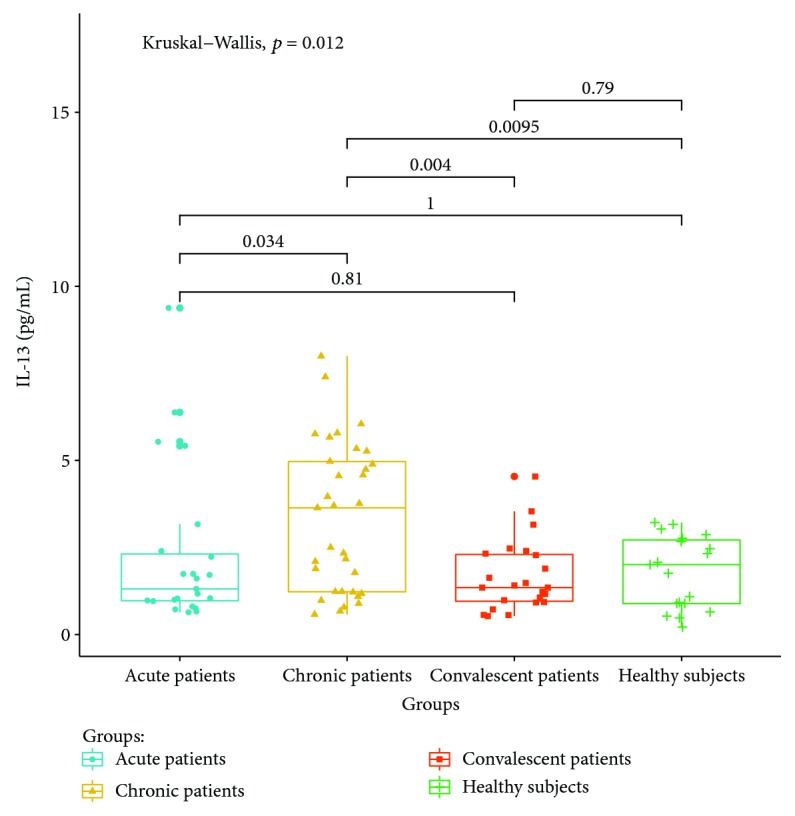
Expression of IL-13A in the peripheral blood of different groups.

**Figure 13:**
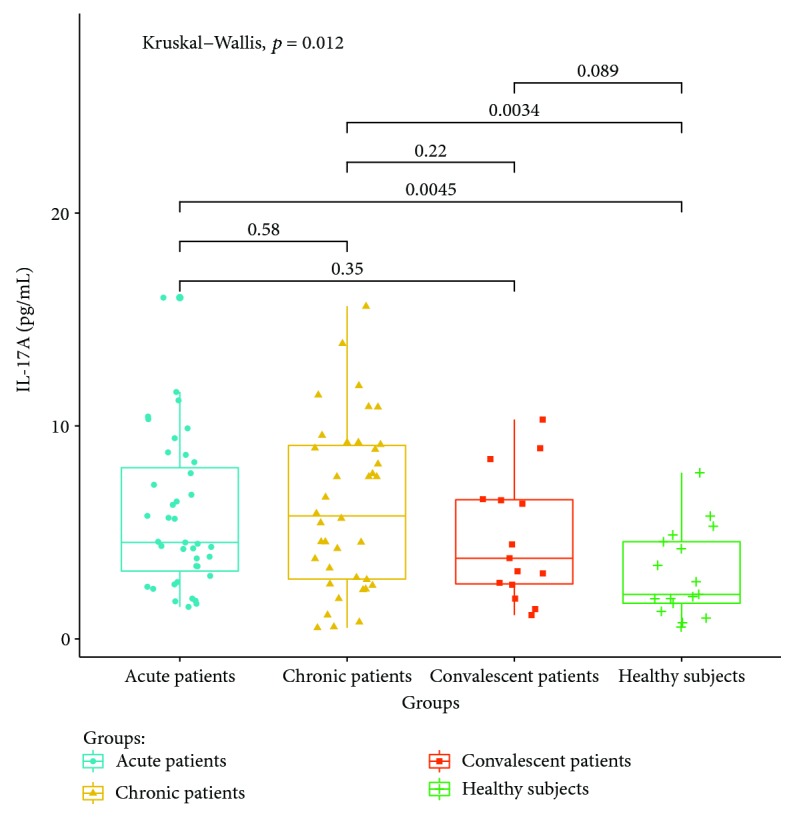
Expression of IL-17A in the peripheral blood of different groups.

**Figure 14:**
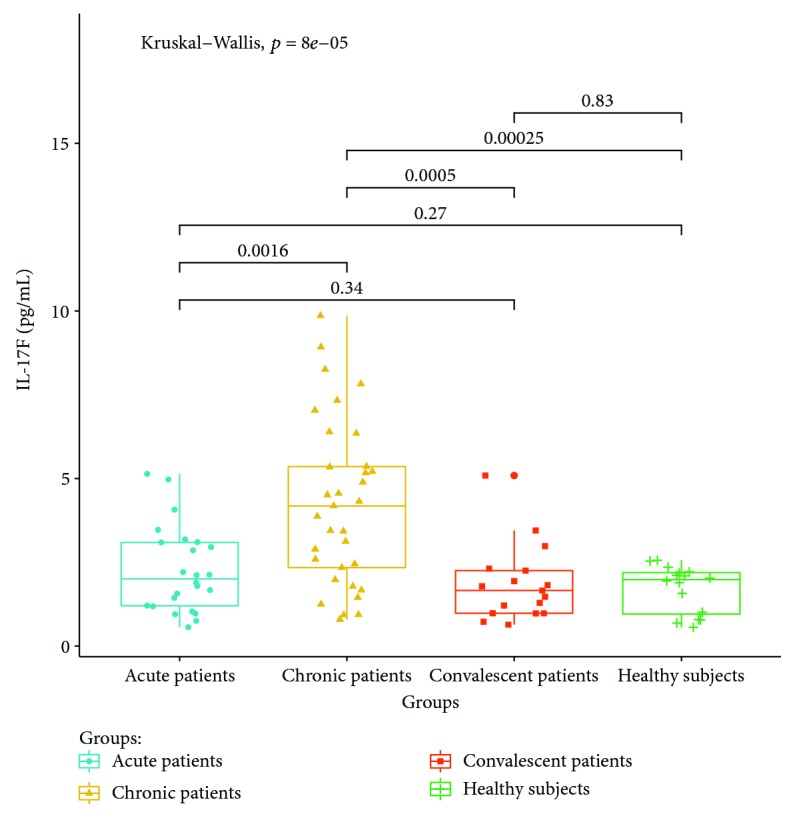
Expression of IL-17F in the peripheral blood of different groups.

Cytokines related to different T lymphocyte subsets in the peripheral serum of patients and healthy controls were detected by cytometric bead analysis. The following results were shown: (1) The expressions of Th1 cell-related cytokine IL-2 in acute and convalescent patients were higher than that in healthy controls (*P* < 0.05). The expression levels had no significant differences between acute, chronic, and convalescent patients (*P* > 0.05). The expression of IL-2 is shown in Figure 8. The expression levels of IFN-gamma in patients at the acute stage were significantly higher than those in patients from the chronic, convalescent, and healthy control groups (*P* < 0.05). The expression levels between patients from the chronic, convalescent, and healthy control groups (*P* > 0.05) had no significant differences. The expression of IFN-gamma is shown in Figure 9. (2) The expression of cytokines was mainly secreted by Th2 lymphocytes. The expressions of IL-4 in chronic and convalescent patients were significantly higher than those in acute and convalescent patients (*P* < 0.05). There were no significant differences between chronic and convalescent patients (*P* > 0.05). The expressions of IL-4 in acute patients were higher than those in healthy patients (*P* > 0.05). The expression of IL-4 is shown in Figure 10. The expression levels of cytokine IL-5 in patients at the chronic stage were significantly higher than those in patients from the acute, convalescent, and healthy control groups (*P* < 0.05). The expression level had no differences between patients from the acute, convalescent, and healthy control groups (*P* > 0.05). The expression of IL-5 is shown in Figure 11. The expression levels of cytokine IL-13 in patients at the chronic stage were higher than those in patients from the acute, convalescent, and healthy control groups (*P* < 0.05), but there were no differences among patients from the acute, convalescent, and healthy control groups (*P* > 0.05). The expression of IL-13 is shown in Figure 12. (3) The expressions of IL-17A, a cytokine secreted mainly by the Th17 lymphocyte, in patients at the acute, chronic, and convalescent stages were higher than those in the healthy control group (*P* < 0.05), but there were no differences in the expression of IL-17A in patients at different stages (*P* > 0.05). The expression of IL-17A is shown in Figure 13. The expression levels of IL-17F in patients at the chronic stage were the highest, significantly higher than those in patients from the acute, convalescence, and healthy control groups (*P* < 0.05). Expression of IL-17F is shown in Figure 14.

## 4. Discussion

At present, the pathogenesis of brucellosis has not been fully understood. And T cell immune response is involved in the whole course of *Brucella* infection. Thus, it is speculated that the immune function of patients at the chronic phase is different from that of patients at the acute phase. By analyzing T cell subsets of patients at different stages of infection, we could reveal the immune system response characteristics of the host after *Brucella* infection and provide theoretical basis for evaluating curative effects and guiding the establishment of clinical staging indicators in the future.

The results show that the expression of Th1 cells in the peripheral blood of patients with brucellosis is significantly higher than that of healthy controls, and the expression level in patients at the acute stage is higher than that in patients at the chronic stage. Previous reports on the expression of Th1 cells in the peripheral blood of patients were controversial [19, 20]. The results of this study suggest that Th1 cells are the main type of cellular immunity at the acute stage of *Brucella* infection, meaning that Th1 cells might play an important role in the prevention of *Brucella* infection. At the same time, we find that the proportion of Th1 cells in convalescent patients is still high, suggesting that although the symptoms are cured after treatment, the recovery of the immune function lags behind the clinical manifestations. Monitoring the immune function of convalescent patients can help to evaluate the patient's condition and curative effect.

Th1 cells mainly secrete interferon gamma and IL-2. The results show that the expression of cytokine IL-2 in the peripheral serum of patients at the acute stage is the highest, which is significantly different from that of healthy people. The expression of IL-2 in convalescent patients after standard anti-*Brucella* treatment is still high, which is consistent with the results of Th1 cells detected in this study. It may be speculated that Th1 cells could play an immune response against *Brucella* infection by increasing the secretion of IL-2, while it is confirmed from another aspect that the recovery of the Th1 cell phenotype and its function in the peripheral blood of convalescent patients lag behind the remission of clinical symptoms. It is necessary to follow up the patients after treatment for a longer time in the future.

The mechanism of IFN-gamma in brucellosis infection is not completely clear yet. According to the results of this study, the secretion of IFN-gamma is upregulated in patients with *Brucella* infection at the acute stage, and it is decreased in patients at the chronic stage or after cure. This is consistent with some previous studies which reported that the level of Th1-related cytokines in the serum of patients with acute brucellosis increased significantly, especially the expression of IFN-gamma. After treatment with antibiotics, all cytokine levels have decreased or even returned to normal [21], which means that IFN-gamma plays a role in resisting *Brucella* infection.

The main cytokines secreted by Th2 cells are interleukin-4 (IL-4), interleukin-5 (IL-5), and interleukin-13 (IL-13) [22]. The results of this study show that the expression rates of Th2 cells in the peripheral blood of patients with chronic brucellosis are the highest, which are significantly higher than those of patients with acute brucellosis. Consistent with the current view, Th2 cells are involved in the chronicity of brucellosis. Different from the Th1 cells observed in this study, the Th2 cells in patients with effective treatment are basically normal, suggesting that Th2 cells may be used as one of the indicators to judge the efficacy and condition of the disease in the future. Some of the previous studies were inconsistent with our conclusions [19, 20]. In view of the fact that the same results could not be obtained, the reasons may be related to the different observer population, treatment status, and disease-staging criteria.

We find that the expression of IL-4 is the highest in chronic and convalescent patients, which is significantly higher than that in acute patients. The increased expression of IL-4 in chronic patients is consistent with the change of Th2, suggesting that IL-4 is involved in the chronicity of *Brucella* infection [21]. IL-13 and IL-5 are typical Th2 cytokines. The results of this study show that the expressions of IL-13 and IL-5 in chronic patients are significantly higher than those in the acute, convalescent, and healthy control groups, suggesting that both IL-13 and IL-5 are involved in the progression of Th2 cell-mediated brucellosis progressing into the chronic phase.

Th17 cells are important immune cells associated with infection [23]. The results show that there are no significant differences in the cell proportions between patients at acute and chronic stages, which are higher than those of healthy controls. This is consistent with Sofian et al.'s report that the percentage of Th17 cells in the peripheral blood of patients with acute brucellosis increased significantly and decreased after treatment [24]. These results suggest that Th17 cells can differentiate and proliferate after *Brucella* infection. It is believed that Th17 cells may participate in the cellular immunity against *Brucella* infection, but the specific mechanism needs further study.

IL-17A and IL-17F are the main effector molecules of Th17 cells [25–29]. The expression of IL-17A in patients with brucellosis is higher than that in healthy controls. The expression levels of IL-17A in convalescent patients are still higher than those in healthy controls. It is suggested that IL-17A may play a role in the clearance of *Brucella* infection, and the expression level of IL-17A does not return to normal in a short time after clinical cure, suggesting that the recovery of the expression level of IL-17A lags behind clinical symptoms. The expression of IL-17F is the highest in chronic patients, suggesting that IL-17F may be involved in the chronicity of *Brucella* infection. Although both IL-17A and IL-17F are cytokines mainly secreted by Th17 cells, the expression of IL-17A and IL-17F in patients at different stages is not consistent, suggesting that they may play different roles in regulating the immune mechanism of brucellosis.

Studies on the pathogenesis of PD-1 in infections indicate that PD-1 plays a role in mediating the progress of infection into chronicity [15, 30–33]. The results of this study show that the expressions of CD4+ and CD8+ T cells in patients at the acute stage are the highest, which are significantly higher than those in patients with chronic brucellosis. It is suggested that the expression of PD-1 on T lymphocytes in the peripheral blood of patients with brucellosis is upregulated. It is speculated that PD-1 is involved in the regulation of immune cell function by *Brucella* infection. At present, PD-1 antagonists are widely used in the study of cancer treatment. Further research is needed to determine whether blocking PD-1 can be used in the diagnosis and treatment of brucellosis, especially for chronic refractory brucellosis, and to evaluate whether PD-1 can be used as an auxiliary diagnostic tool to judge the efficacy of treatment.

Previous reports showed that *Brucella* could be cleared more effectively when Treg cells were neutralized by antibodies [34]. The results show that the expressions of Treg cells in the peripheral blood of patients at acute and chronic stages are higher than those of the healthy control group, but there are no differences between patients at the acute and chronic stages. This is inconsistent with the findings of Skendros et al. who have reported that the percentage of CD4+ CD25+ T lymphocytes in the peripheral blood of patients with chronic recurrent brucellosis was lower than that of patients with acute brucellosis [35]. The inconsistency may result from the different stages and the course of the disease. The results of this study show that Treg cells continue to increase in chronic patients, suggesting that Treg cells play a certain role in the chronicity of *Brucella* infection.

Our study has this limitation: we did not observe the dynamic changes of these routine parameters and levels of T cell subsets and cytokine in the course of drug treatment.

In summary, Th1, Th17, and Treg cell immunity are predominant in the acute phase and Th2, Th17, and Treg cell immunity predominated in the chronic phase after *Brucella* infection, suggesting that Th2, Th17, and Treg cells may be involved in the chronicity of *Brucella* infection. The increased expression of PD-1 on the T lymphocyte surface in patients with brucellosis suggests that PD-1 may play a role in the pathogenesis of brucellosis, and further studies about this are needed. The immune function of the patients is not completely restored in a short time after clinical cure, and the patients in the convalescent period need long-term follow-up. In the future, biochemical or immunological indicators that can accurately assess the condition, efficacy, and prognosis of the disease need to be found by high-quality clinical research.

## Figures and Tables

**Table 1:** Clinical characteristics of acute, chronic, and convalescent patients.

Clinical manifestations	Acute stage (*n* = 47)	Chronic stage (*n* = 43)	Convalescent stage (*n* = 35)
Fever	46 (97.9%)	39 (90.7%)	0
Fatigue	40 (85.1%)	41 (95.3%)	3 (8.6%)
Chills	20 (42.6%)	5 (11.6%)	0
Sweats	30 (63.8%)	10 (23.3%)	1 (2.9%)
Joint pain	39 (83%)	35 (81.4%)	2 (5.7%)
Headache	15 (31.9%)	5 (11.6%)	0
Muscle pain	40 (85.1%)	20 (46.5%)	0
Weight loss	9 (21.3%)	2 (4.7%)	0
Cough	7 (14.9%)	0	0
Orchitis/epididymitis	5 (14.7%)	2 (7.7%)	0

**Table 2:** Laboratory characteristics of acute, chronic, and convalescent patients.

Laboratory examinations	Acute stage (*n* = 47)	Chronic stage (*n* = 43)	Convalescent stage (*n* = 35)
Positive erythrocyte plate test	47 (100%)	43 (100%)	35 (100%)
Positive blood culture	7 (14.9%)	0	0
Leucocytopenia	2 (4.3%)	3 (7%)	0
Hyperleukocytosis	4 (8.5%)	0	0
Anemia	6 (12.8%)	8 (18.6%)	0
Thrombocytopenia	2 (4.3%)	5 (11.6%)	0
Increased erythrocyte sedimentation rate	40 (85.1%)	28 (65.1%)	0
Increased C reactive protein	34 (72.3%)	26 (60.5%)	0
Enlargement of liver, spleen, and (or) lymph nodes	15 (31.9%)	16 (37.2%)	0
Abnormal liver function	13 (27.7%)	3 (7%)	0
Abnormal urine routine	17 (36.2%)	11 (25.6%)	0
Abnormal chest X-ray	8 (17%)	2 (4.7%)	0

**Table 3:** Correlation between T lymphocyte subsets and withdrawal time in convalescent patients.

	Withdrawal time	Th1	Th2	Th17	Treg
Withdrawal time	1.000				
Th1	-0.679^∗∗^	1.000			
Th2	0.056	-0.232	1.000		
Th17	-0.168	0.036	0.109	1.000	
Treg	-0.256	0.088	0.047	-0.136	1.000

Note. ^∗^Represents significant correlation at 0.05 level. ^∗∗^Represents significant correlation at 0.01 level.

## Data Availability

The data used and/or analyzed in the current study are available from the corresponding author on reasonable request.
